# APSIC guide for prevention of catheter associated urinary tract infections (CAUTIs)

**DOI:** 10.1186/s13756-023-01254-8

**Published:** 2023-05-30

**Authors:** Moi Lin Ling, P. Ching, A. Apisarnthanarak, N. Jaggi, G. Harrington, S. M. Fong

**Affiliations:** 1 Infection Prevention and Epidemiology, Singapore General Hospital, Outram Road, Singapore, 169403 Singapore; 2 The University of Hong Kong, Pok Fu Lam, Hong Kong China; 3 Thammasat University Hospital, Khlong Luang, Thailand; 4 Artemis Hospital, Gurgaon, India; 5 Infection Control Consultancy, Melbourne, Australia; 6 Sabah Women and Children’s Hospital, Kota Kinabalu, Malaysia

**Keywords:** Catheter associated urinary tract infection, CAUTI, Prevention, Infection control

## Abstract

**Background:**

The Asia Pacific Society of Infection Control launched the APSIC guide for prevention of catheter associated urinary tract infections in July 2022. It aims to highlight practical recommendations in a concise format designed to assist healthcare facilities in the Asia Pacific region to achieve high standards in infection prevention and control practices during the management and care of patients with a urinary catheter.

**Methods:**

The guidelines were developed by an appointed workgroup comprising experts in the Asia Pacific region, following reviews of previously published guidelines and recommendations relevant to each section.

**Results:**

It recommends that healthcare institutions have a catheter associated urinary tract infection prevention program that includes surveillance and the use of the insertion and maintenance bundles. Implementation of the bundles is best done using a quality improvement approach with a multidisciplinary team.

**Conclusions:**

Healthcare facilities should aim for excellence in care of patients with urinary catheters. It is recommended that healthcare facilities have a catheter associated urinary tract infection prevention program as part of their Infection Prevention and Control program.

## Introduction

This document is a summary of the APSIC guide for prevention of catheter associated urinary tract infections (CAUTIs) developed by the Asia Pacific Society of Infection Control (APSIC) to provide the user an overview of its content The full APSIC guide for prevention of catheter associated urinary tract infections is available at https://aspic-apac.org should be read and used as reference to guide practice.

## Review Workgroup Composition

APSIC convened experts in Infection Prevention and Control from the Asia Pacific region to develop the APSIC guide for prevention of catheter associated urinary tract infections (CAUTIs). The members of this workgroup are the authors of this paper.

## Literature Review and Analysis

For the APSIC guideline, the workgroup reviewed previously published guidelines and recommendations relevant to each section and performed computerized literature searches using PubMed. Some examples of keywords used in search include catheter associated urinary tract infection, CAUTI, infection prevention and control.

## Process

The workgroup met on four occasions in addition to discussion via email correspondences to complete the development of the guideline. Criteria for grading the strength of recommendation and quality of evidence are described in Table 1. The draft was then submitted to two external reviewers, APSIC Executive Committee and national Infection Prevention & Control societies in Asia Pacific. Comments obtained were then reviewed by the workgroup for necessary edits, following which the final draft was circulated for approval and endorsement by the APSIC Executive Committee and national societies from the Asia Pacific region.

**Table 1:** Categories for strength of each recommendation

Category	Definition
*Categories for strength of each recommendation*	
A	Good evidence to support a recommendation for use
B	Moderate evidence to support a recommendation for use
C	Insufficient evidence to support a recommendation for or against use
D	Moderate evidence to support a recommendation against use
E	Good evidence to support a recommendation against use

Grade	Definition
*Categories for quality of evidence on which recommendations are made*	
I	Evidence from at least one properly randomized, controlled trial
II	Evidence from at least one well-designed clinical trial without randomization, from cohort or case-controlled analytic studies, preferably from more than one centre, from multiple time series, or from dramatic results in uncontrolled experiments
III	Evidence from opinions of respected authorities on the basis of clinical experience, descriptive studies, or reports of expert committees

## Epidemiology of catheter associated urinary tract infection

Among urinary tract infections (UTIs) acquired in the hospital, 75% are catheter-associated UTIs (CAUTI) [1]. It is estimated that more than 25% of hospitalized patients had urinary catheter placement during their hospital stay [1, 2]. Catheter associated urinary tract infection is the most common type of healthcare associated infections (HAI) in the United States [2] and is the second most common HAI in Southeast Asia [3]. Based on a systematic review from high-income versus low-income countries, the cumulative incidence of CAUTIs was estimated to be 4.1 versus 8.8 per 1000 catheter-days [4]. It is well recognized that catheterization and the duration of catheterization is a predictor for CAUTI. Other risk factors for CAUTIs include catheterization outside the operating room, female sex, underlying illness, older age, microbial colonization of drainage bag, catheter and periurethral segment of the catheter [5, 6]. In a CAUTI prevention program, risk factors are to be evaluated and appropriate measures implemented to mitigate the risks. Risks include urinary catheters being inserted unnecessarily, catheters remaining in place for too long, poor aseptic technique during insertion and catheter maintenance, failure to maintain a closed drainage system, obstructed urine flow and no or poor catheter securement.

Recommendations for initial steps to the prevention of CAUTI include

1. Ensure staff have an understanding of the pathogenesis and risk factors for CAUTIs in order to develop and implement appropriate infection prevention measures. [IA]
2. Undertaking risk assessments will identify modifiable risk factors to reduce CAUTI. [IA]

## Diagnosis of CAUTI [7–12]

Generally, the diagnosis of CAUTI is made based on the presence of bacteriuria with signs and symptoms consistent with UTI in a catheterized patient or in a patient who had the catheter removed within the past 48 h. Signs and symptoms compatible with CAUTI include new onset or worsening of fever, rigors, altered mental status, malaise or lethargy. Local symptoms include flank pain, costovertebral angle tenderness, acute hematuria, pelvic discomfort and in those whose catheters have been removed, dysuria, urgent or frequent urination, or suprapubic pain or tenderness may occur.

Bacteriological urine culture is essential for the diagnosis of CAUTI. Urine collection for culture must be done using aseptic technique to avoid contamination and unnecessary antimicrobial treatment. Ideally urine samples for culture should be obtained by mid-stream collection after removal of the indwelling urine catheter. In patients with a long-term indwelling catheter where removal is not possible, the preferred method of obtaining a urine specimen for culture is to replace the catheter and collect the specimen from the freshly placed catheter. Alternatively, urine should be obtained from the catheter access port in the drainage system. The collection port must be disinfected with an appropriate disinfectant such as 70% alcohol and allow to dry for at least 30 s prior to accessing the port. Urine culture should not be taken directly from the drainage bag.

Recommendations for diagnosis of CAUTI include

1. Microbiological confirmation of urine culture is needed for diagnosis of CAUTI. Urine culture must have no more than 2 species of organisms, at least one of which has bacterial growth of > 10.^5^ CFU/ml. [IA]
2. Urine collection for culture must be done using aseptic technique to avoid contamination. [IA]

## Catheter associated urinary tract infection (CAUTI) prevention program [13–15]

The primary outcomes for CAUTI prevention programs are to reduce unnecessary catheter placement and minimize the duration catheter remainis in place. Organizations should develop CAUTI prevention programs that ensure:

- Provision of evidence-based guidelines for catheter use, insertion, and maintenance
- Staff education and periodic training in insertion and removal technique, maintenance procedures, complications, infection prevention and alternatives to indwelling urinary catheters
- Supervised practice and competency assessment of staff to ensure only trained and competent staff insert urinary catheters
- Adequate supplies and equipment to ensure aseptic technique during catheter insertion and maintenance requirements
- Documentation systems that record medical order for catheter placement, indications for catheter insertion, name of person inserting catheter, date, time, daily maintenance care and assessment of the ongoing need for catheter and planned date of removal
- Adequately trained staff to support surveillance and feedback of catheter use and outcomes
- Where surveillance identifies opportunities for improvement, support for the implementation of evidence prevention strategies with ongoing surveillance and feedback.

Recommendation:

1. A CAUTI prevention program should be developed [IA]

## Surveillance [16, 17]

Surveillance of CAUTI is important to monitor trends for detection of outbreaks and the assessment of efficacy of CAUTI prevention programs. Performing surveillance for CAUTI is best based on a facility’s risk assessment and/or local regulatory requirements. The facility should consider identifying patient groups or units at risk for CAUTI through reviews on frequency of urinary catheter use and other potential risk factors, such as genitourinary surgery, obstetrics, critical care to develop a targeted surveillance program.

Infection Prevention and Control (IPC) leadership is responsible for ensuring that an active program to identify HAIs is implemented and that HAI data are analyzed with timely feedback to those who can use the information to improve the quality of care (e.g., Heads of units, unit nursing staff, clinicians, and hospital administrators), and that evidence-based practices are incorporated into the program.

Recommendations for surveillance of CAUTI include:

1. Use standardized methodology for performing CAUTI surveillance [IA]
2. Routine screening of catheterized patients for asymptomatic bacteriuria is not recommended. [IIB]
3. When performing surveillance for CAUTI, regular feedback of unit specific CAUTI rates to nursing staff and clinical care staff. [IIB]
4. Reporting of outcome measures to senior administrative, medical, and nursing leadership and clinicians who care for patients at risk for CAUTI. [IIB]

## Implementing a catheter associated urinary tract infection (CAUTI) prevention program [18–21]

Successful results have been seen when healthcare institutions adopt a quality improvement approach to reduce healthcare associated infections (HAIs). The Comprehensive Unit-based Safety Program (CUSP) is one successful example [1]. A multidisciplinary team comprising key stakeholders as team members should work together in reviewing local issues and adopting a Plan-Do-Study-Act (PDSA) approach to address the issues [2]. The model for improvement recommended is described in the APSIC guide for prevention of central line associated bloodstream infections (CLABSI). Changes are made in rapid PDSA cycles by a multidisciplinary team.

Recommendation:

1. Implementation of CAUTI insertion and maintenance bundles is best done using a quality improvement approach with a multidisciplinary team. [IA]

## Conclusion

We recommend healthcare facilities to have a CAUTI prevention program as part of their IPC program.

*Endorsed by*:

1. Hong Kong Infection Control Nurses Association (HKICNA), Hong Kong, China
2. Ho Chi Minh City Infection Control Society (HICS), Vietnam
3. Indonesian Society of Infection Control (INASIC), Indonesia
4. Infection Control Association (ICAS), Singapore
5. Persatuan Kawalan Infeksi dan Antimikrobial Kota Kinabalu Sabah (PKIAKKS), Borneo, Malaysia
6. Malaysian Society of Infection Control and Infectious Diseases (MyICID), Malaysia
7. National Nosocomial Infection Control Group (NNIG), Thailand
8. Vietnam National Infection Control Society (VNICS), Vietnam

## Data Availability

Yes; the APSIC guide for prevention of catheter associated urinary tract infections (CAUTIs) is available at the APSIC website (aspic-apac.org).
